# Diabetic Peripheral Neuropathy: Current Epidemiology, Diagnostic Advances, Biomarkers, and Management Strategies

**DOI:** 10.1155/jdr/9080699

**Published:** 2026-04-21

**Authors:** Felix T. Kurz, Johann M. Jende, Rares Salomir, Karl-Olof Lövblad, Agustina Lascano, Karim Gariani

**Affiliations:** ^1^ Department of Neuroradiology, Geneva University Hospitals, Geneva, Switzerland, hug-ge.ch; ^2^ Division of Radiology, German Cancer Research Center, Heidelberg, Germany, dkfz.de; ^3^ Division of Radiology, Diagnostic Department, Geneva University Hospital, 1211, Geneva, Switzerland, hug-ge.ch; ^4^ Image Guided Interventions Laboratory (GR-949), Faculty of Medicine, University of Geneva, 1211, Geneva, Switzerland, unige.ch; ^5^ Department of Clinical Neurosciences, Division of Neurology, Geneva University Hospitals and Faculty of Medicine, University of Geneva, Geneva, Switzerland, unige.ch; ^6^ Division of Endocrinology, Diabetes and Metabolism, Department of Medical Specialties, Geneva University Hospitals, Geneva, 1205, Switzerland, hug-ge.ch; ^7^ Faculty Diabetes Center, University of Geneva Medical Center, University of Geneva, Geneva, Switzerland, unige.ch

**Keywords:** autonomic dysfunction, diabetes, diabetes-related complications, diabetic neuropathy, diagnostic tools, imaging, magnetic resonance imaging, neuropathic pain, small fiber neuropathy

## Abstract

Diabetic peripheral neuropathy (DPN) is one of the most common and disabling complications of diabetes mellitus (DM), affecting up to 50% of patients during the course of their disease. The condition manifests predominantly as a distal symmetric polyneuropathy, with early involvement of small fibers and progressive sensory loss. Epidemiological data show high prevalence not only in type 1 and type 2 diabetes but also in prediabetic populations, highlighting the need for early detection. The pathophysiology of DPN is complex, involving metabolic dysregulation, oxidative stress, vascular insufficiency, inflammation, and mitochondrial dysfunction. Diagnostic tools range from clinical examination and nerve conduction studies (NCS) to magnetic resonance imaging. Recent advances in plasma and neuroimaging biomarkers offer promising avenues for earlier and more accurate diagnosis. Management focuses on strict glycemic control, risk factor modification, symptomatic treatment of neuropathic pain, and lifestyle interventions. Despite ongoing research, no curative therapy currently exists, underscoring the urgent need for disease‐modifying strategies. A multidisciplinary, patient‐centered approach is essential to mitigate complications and improve quality of life. This review provides a comprehensive overview of DPN, covering its epidemiology, pathogenesis, diagnostic approaches, emerging biomarkers, and current management strategies. Future research should emphasize biomarker validation, longitudinal monitoring, and personalized therapeutic interventions.

## 1. Introduction

The worldwide surge in the incidence of prediabetes and diabetes mellitus (DM) has been paralleled by a marked increase in the associated disorders [1, 2]. Among these, diabetic peripheral neuropathy (DPN) represents the most prevalent chronic complication, with distal symmetric polyneuropathy emerging as the predominant clinical manifestation. DPNDPN encompasses a broad clinical spectrum ranging from painless forms, characterized by sensory loss (e.g., numbness, reduced vibration, and thermal perception), to painful forms (painful DPN [pDPN]) which affect ~15%–25% of patients and are characterized by neuropathic pain arising from damage to the somatosensory nervous system (for a definition of neuropathic pain see Finnerup et al.) [3]. Neuropathic pain is typically described as burning, shooting, electric shock‐like or stabbing in nature and may be accompanied by allodynia or hyperalgesia. In contrast, individuals with painless DPN often lack overt pain despite significant nerve dysfunction, placing them at increased risk of unrecognized injuries and foot complications [4]. When autonomic neuropathy is present, the autonomic nervous system fails to regulate essential physiological functions effectively, resulting in significant impairments, especially in cardiovascular and gastrointestinal regulation [5].

DPN represents a significant public health concern, primarily due to its steadily increasing prevalence and its substantial negative impact on patients’ quality of life [6]. As the number of individuals affected by diabetes continues to rise worldwide, the burden of DPN is expected to grow accordingly. Beyond its physical symptoms, DPN is associated with chronic pain, reduced mobility, sleep disturbances, and psychological distress, all of which contribute to a marked deterioration in overall well‐being and daily functioning [7].

In this review, we provide a comprehensive overview of current knowledge on DPN, including its epidemiology, pathogenesis, diagnostic approaches, and both current and emerging treatment strategies. A literature search was performed in PubMed to identify relevant studies on DPN. The search strategy combined keywords and Medical Subject Headings (MeSH) terms, including “diabetes mellitus,” “diabetic peripheral neuropathy,” “painful diabetic neuropathy,” “diagnosis,” “biomarkers,” “nerve conduction studies,” “magnetic resonance imaging,” and “management.” The literature search was conducted from inception to October 2025, with priority given to systematic reviews and clinical trials published in English, and additional references were identified through the reference lists of selected publications.

## 2. Epidemiology

DPN represents the predominant subtype of neuropathic disorders globally, both in prevalence and clinical incidence. Epidemiological data indicate that the prevalence of DPN among adults with DM varies widely, ranging from around 5% to more than 50% [1], with more recent analyses suggesting an upward trend in these estimates [8].

Extensive longitudinal studies have contributed to a clearer understanding of the prevalence and progression of DPN in 1441 individuals with type 1 diabetes across different age groups. In the historical DCCT/EDIC cohort, 6% of adult participants exhibited peripheral neuropathy at baseline, with the prevalence rising to nearly one‐third after 4 years of follow‐up, highlighting the gradual and progressive nature of the condition [9, 10]. Similarly, the Pittsburgh Epidemiology of Diabetes Complications study reported a 34% prevalence of DPN among 400 type 1 diabetic adults, with a marked increase associated with age: 18% in individuals aged 18–29 years and 58% among those aged 30 years and older [11]. In younger type 1 diabetic populations, data from the SEARCH for Diabetes in Youth Study revealed a lower prevalence of around 8% among the 329 participants with a mean age of 16 years old [12, 13]. Collectively, these findings underscore the age‐related increase in DPN prevalence and emphasize the need for early detection and long‐term surveillance in patients with type 1 diabetes.

The burden of DPN appears to be greater among individuals with type 2 diabetes compared to those with type 1. In adult populations, baseline data from the ACCORD trial including more than 10,000 partcipants indicated a prevalence of 42% for DPN [14]. Similarly, findings from the BARI 2D trial showed that over half of adults with type 2 diabetes reported a prior diagnosis of peripheral neuropathy at study entry [15]. Recent studies report varying prevalence estimates for DPN. The prevalence of possible DPN ranges from 22.1% to 35.0%, while pDPN is reported between 14.5% and 20.0%. In some large cohorts of patients with type 2 diabetes and DPN, over half (57.2%) were affected by painful neuropathy [16–18].

In younger population, evidence from the SEARCH for Diabetes in Youth Study revealed that approximately a quarter of adolescents diagnosed with type 2 diabetes exhibited signs of peripheral neuropathy [12]. These observations underscore the high prevalence of DPN in type 2 diabetes and highlight its early onset in youth as well as its substantial presence in adults at the time of clinical evaluation.

Prediabetes is defined by elevated blood glucose levels that fall between normoglycemia and the diagnostic threshold for type 2 diabetes. As of 2021, an estimated 10.6% of the global adult population had impaired glucose tolerance, while 6.2% exhibited impaired fasting glucose [1]. A substantial proportion of these individuals remain undiagnosed. Emerging evidence suggests a higher prevalence of peripheral neuropathy among individuals with prediabetes. A meta‐analysis comprising 29 studies and a total of 9351 participants demonstrated considerable heterogeneity in prevalence estimates. Furthermore, the underlying disease trajectory of DPN has not been fully clarified, since the respective contributions and temporal sequence of small‐ and large‐fiber involvement remain inadequately understood [19]. These neuropathies, predominantly attributed to small fiber dysfunction, appear significantly more common in the prediabetic population than in the general population. Given the rising global burden of prediabetes, there is a growing imperative to consider targeted screening strategies within this at‐risk group. Altogether, epidemiological findings underscore that distal peripheral neuropathy is not exclusively associated with overt diabetes, but is also observed at a meaningful prevalence in individuals with prediabetes, suggesting an earlier onset of nerve involvement in the glycemic continuum. Further high‐quality longitudinal and interventional studies are essential to identify risk factors associated with neuropathy development in prediabetes and to assess the potential benefits of lifestyle‐based preventive interventions.

## 3. Pathogenesis

Despite extensive research, the pathogenetic mechanisms of DPN remain only partially understood, reflecting a multifactorial process involving metabolic, vascular, inflammatory, and neurotrophic imbalances. DPN is clinically characterized by a length‐dependent, symmetrical neuropathy that predominantly affects distal limbs, with symptoms ranging from pain and paresthesia to numbness and motor dysfunction. Its development involves both functional and structural alterations of peripheral nerves, along with critical damage to glial support cells such as Schwann cells [20].

**Figure 1:**
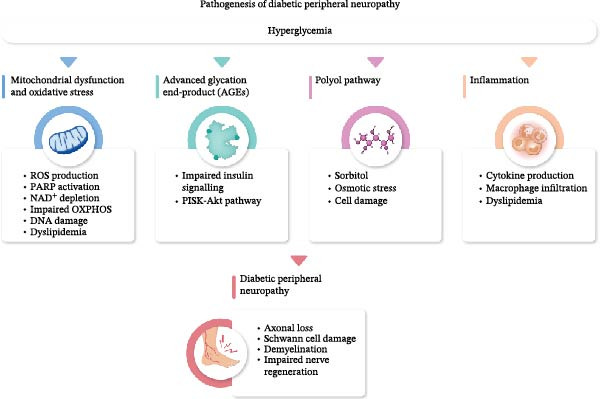
Pathogenesis of diabetic peripheral neuropathy. Chronic hyperglycemia triggers multiple pathways contributing to diabetic peripheral neuropathy, including mitochondrial dysfunction and oxidative stress, accumulation of advanced glycation end‐products (AGEs), activation of the polyol pathway, and inflammation. These mechanisms lead to axonal loss, Schwann cell damage, demyelination, and impaired nerve regeneration.

Hyperglycemia and dyslipidemia are central metabolic drivers of DPN, triggering a cascade of intracellular disturbances. Excess glucose influx into alternative metabolic pathways induces oxidative and nitrosative stress, impairs mitochondrial function, and contributes to progressive axonal degeneration and demyelination. The accumulation of sorbitol via the polyol pathway, the formation of advanced glycation end‐products (AGEs), and the activation of signaling molecules such as protein kinase C (PKC) contribute to osmotic stress, inflammatory responses, and vascular dysfunction [21]. Although some pathways, such as PKC, can exert both neuroprotective and detrimental effects depending on the cellular context, their sustained activation in the diabetic milieu generally promotes neurodegeneration (Figure 1).

Alterations in insulin signaling are also increasingly recognized as contributing to DPN. Insulin plays a critical neurotrophic role beyond its glycemic regulatory function, supporting axonal growth, myelin integrity, and Schwann cell survival. In diabetes, insulin resistance or deficiency disrupts these signaling pathways, particularly the PI3K‐Akt signaling pathway, resulting in impaired axonal maintenance, myelin loss, and defective nerve conduction [22].

Concurrently, DNA damage induced by oxidative stress activates poly(ADP‐ribose) polymerase (PARP), leading to depletion of nicotinamide adenine dinucleotide (NAD^+^) and ATP and further impairing cellular energetics and mitochondrial health. The resultant energy deficit compromises axonal transport and repair mechanisms, reinforcing the degenerative process [23]. The hexosamine biosynthetic pathway and associated modifications of transcription factors such as Sp1 additionally promote the expression of pro‐inflammatory and fibrotic mediators, further contributing to the hostile metabolic environment [24].

Structural damage in DPN encompasses both demyelination and axonal loss, involving both small unmyelinated fibers and large myelinated fibers. Schwann cells, essential for nerve insulation and metabolic support, are particularly vulnerable to hyperglycemic stress and undergo early dysfunction. Microangiopathy, including basement membrane thickening, endothelial cell loss, and impaired autoregulation of blood flow, exacerbates ischemic injury and impairs nerve regeneration [20]. Furthermore, mitochondrial dysfunction plays a key role in the pathogenesis of DPN. Chronic hyperglycemia disrupts oxidative phosphorylation, shifting energy production toward anaerobic metabolism and compromising the high metabolic demands of peripheral neurons, particularly those with long axons [25].

Inflammatory processes intersect with these metabolic and vascular changes, with cytokine production, immune cell infiltration, and altered neuroimmune signaling reinforcing neural injury. Impaired neurotrophin expression and disturbed retrograde axonal transport further diminish the capacity for nerve repair and regeneration.

The pathogenesis of DPN is governed by a complex and interconnected network of metabolic, vascular, and inflammatory pathways, acting on both neurons and glial elements. While considerable progress has been made in identifying key molecular players, the full picture remains incomplete, underscoring the need for continued investigation to develop targeted and effective neuroprotective strategies.

## 4. Clinical Presentation

The clinical characterization of DPN primarily hinges on a comprehensive neurological assessment, as no single biomarker currently offers definitive diagnostic value. DPN manifests through a heterogeneous array of sensory and motor deficits, attributable to the progressive dysfunction of both small and large peripheral nerve fibers. The neuropathic process typically follows a distal‐to‐proximal gradient, initially affecting the most distal segments of the lower extremities, most notably the toes, before advancing proximally in a symmetrical fashion. This distribution pattern results in the classical “stocking‐and‐glove” sensory loss phenotype. Small fiber involvement frequently precedes large fiber degeneration and is commonly associated with positive sensory phenomena such as neuropathic pain; however, the temporal relationship between small‐ and large‐fiber dysfunction is heterogeneous, and small fiber changes do not uniformly precede large fiber degeneration [26–29]. Patients may report a wide spectrum of pain descriptors, including burning sensations, pins and needles, lancinating or stabbing pain, and paroxysmal electric‐shock‐like discharges. In parallel, various forms of sensory distortion may occur, including paresthesias, dysesthesias, allodynia, and hyperalgesia. These clinical features exert a substantial detrimental effect on patients’ quality of life, frequently contributing to sleep impairment and psychological morbidity [30, 31].

As the disease progresses and large‐diameter myelinated fibers are affected, patients typically develop a reduction or complete loss of deep pressure, vibration, and proprioceptive modalities. This leads to sensory ataxia, impaired balance, and gait instability. The absence of protective sensation in the feet predisposes [5] individuals to repetitive unnoticed trauma, which is a major precipitant of foot ulceration. In the context of impaired wound healing and increased susceptibility to infection, these lesions may evolve into severe complications, including osteomyelitis and eventual lower‐limb amputation. Furthermore, deficits in proprioceptive feedback contribute to increased fall risk, particularly in older adults, thereby elevating the incidence of fractures and functional decline. The clinical heterogeneity and progressive nature of DPN underscore the need for early detection and comprehensive multidisciplinary management strategies to mitigate long‐term morbidity.

While clinical features may strongly indicate DPN, maintaining a broad differential diagnosis remains essential, as various neuropathic conditions can coexist in individuals with diabetes and may be reversible with targeted interventions. Potential alternative or contributing etiologies include renal insufficiency [32], thyroid disorders [33], chronic alcohol use, nutritional deficiencies (especially vitamin B12), certain pharmacologic agents (e.g., cisplatine, vincristine, bortézomib, amiodarone, immune checkpoint inhibitor [34]…) chronic inflammatory demyelinating polyradiculoneuropathy (CIDP), systemic amyloidosis, infections, and autoimmune diseases [35].

## 5. Diagnosis

Accurate and timely identification of DPN is essential to mitigate the risk of serious complications, including foot ulceration and limb amputation. Current diagnostic approaches emphasize a multidimensional strategy, integrating clinical evaluation with objective testing modalities tailored to the specific phenotype of neuropathy.

Importantly, several scientific societies recommend a graded diagnostic framework, distinguishing *possible*, *probable*, and *confirmed* DPN, as well as analogous levels of diagnostic certainty for neuropathic pain. A thorough patient history is fundamental for detecting early signs of sensory, motor, or autonomic dysfunction, while the physical examination should systematically assess sensory deficits, muscle strength, and deep tendon reflexes [36]. To improve diagnostic accuracy and reproducibility, clinical guidelines strongly encourage the use of validated scoring systems that integrate both symptoms and neurological signs of DPN. Such composite neuropathy scores, including the Michigan Neuropathy Screening Instrument (MNSI), the Neuropathy Disability Score (NDS), and the Neuropathy Impairment Score (NIS), among others, provide standardized and reproducible measures for the assessment and stratification of DPN [37, 38]. Bedside sensory assessments play a pivotal role in initial screening. These include the use of a 10 g monofilament to evaluate pressure detection thresholds [39], the 128 Hz tuning fork for assessing vibration perception, and thermal as well as pinprick testing to probe small fiber integrity. In parallel, diminished or absent ankle reflexes often signal large fiber involvement. In cases with atypical clinical presentations or diagnostic uncertainty, nerve conduction studies (NCS) are instrumental in differentiating DPN from other etiologies.

While these clinical assessments form the cornerstone of DPN diagnosis, additional neurophysiological, biological, and imaging techniques are often required in cases of diagnostic uncertainty, atypical presentations, or for confirmation and phenotyping of neuropathy. These approaches are discussed in detail below.

### 5.1. NCS as Diagnostic Biomarkers of Neuropathy

Classical NCS has represented a cornerstone of electrophysiological evaluation in DPN, serving as diagnostic, prognostic, and staging tools [40]. These recordings enable the separate assessment of motor and sensory fiber integrity [41] (Table 1).

**Table 1:** Established and emerging strategies for the diagnosis of diabetic peripheral neuropathy.

Established diagnostic approaches	Emerging and future diagnostic approaches
Clinical assessment and validated neuropathy scores (MNSI, NDS, NIS)	Circulating biomarkers reflecting neuroaxonal injury and inflammation (e.g., neurofilament light chain, cytokines, microRNAs)
Bedside sensory testing (10 g monofilament, vibration perception with 128 Hz tuning fork, thermal and pinprick testing)	Advanced electrophysiological techniques assessing small‐fiber pathways (contact heat–evoked potentials, laser‐evoked potentials)
Nerve conduction studies for large‐fiber dysfunction	Corneal confocal microscopy as a non‐invasive biomarker of small‐fiber pathology
Quantitative sensory testing and skin biopsy for small‐fiber assessment	High‐resolution imaging approaches such as magnetic resonance neurography
Autonomic function testing (e.g., sudomotor function using Sudoscan)	Integrated multimodal approaches combining imaging, electrophysiology, and molecular biomarkers

Abbreviations: MNSI, michigan neuropathy screening instrument; NDS, neuropathy disability score; NIS, neuropathy impairment score.

Among the principal electrophysiological parameters, the amplitude of the compound muscle action potential (CMAP) or the sensory nerve action potential (SNAP) is widely regarded as a reliable marker of axonal degeneration. In contrast, indices such as conduction velocity, distal motor latency, and F‐wave latency and duration (defined as the interval between the onset of the initial negative peak and the return to baseline of the final negative peak) are more indicative of demyelination. It is important to note, however, that conduction velocity can also be influenced by axonal diameter, internodal distance, and the biophysical characteristics of the nodal membrane. Abnormalities detected by NCS in diabetic neuropathies typically present with a symmetrical distribution [42] (Figure 2).

Figure 2Nerve conduction studies in an 80‐year‐old male with type 2 diabetes and sensory–motor complaints. (a, b) Sensory nerve conduction studies using antidromic stimulation: (a) sural nerve and (b) radial nerve. (c) Motor conduction study of the peroneal nerve, recorded at the extensor digitorum brevis (EDB) with stimulation at the ankle, below, and above the fibular head. Asterisks ( ^∗^) indicate abnormal results in terms of amplitude and conduction velocity after motor and sensory nerve stimulation. The sural nerve recordings show reduced sensory nerve action potential (SNAP) amplitudes consistent with axonal loss, as well as conduction slowing suggestive of superimposed demyelination (which can be also secondary to axonal degeneration). In contrast, radial nerve SNAPs were within normal range, illustrating a length‐dependent pattern typical of diabetic neuropathy. The peroneal compound muscle action potential (CMAP) demonstrates decreased amplitude compatible with axonal loss and mild slowing of conduction velocity.

**Figure:**
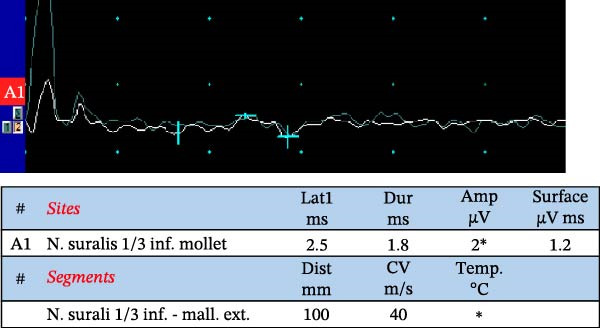
(a)

**Figure:**
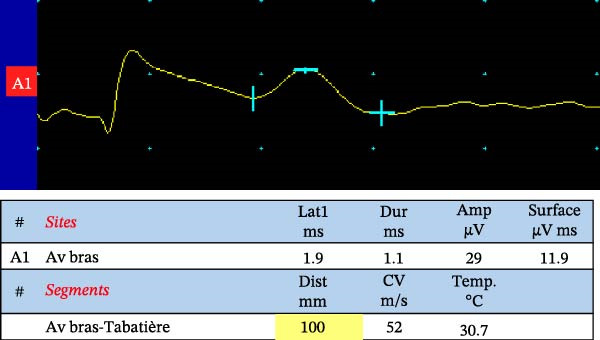
(b)

**Figure:**
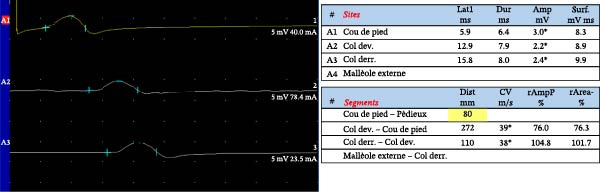
(c)

While NCS are widely accessible and standardized in many clinical settings, their routine application is particularly valuable in cases with atypical presentation (i.e., asymmetric, rapid onset, cranial nerve palsy, amyotrophy, proximal onset) [43, 44]. When performed by experienced clinicians, these tests yield quantitative and reproducible data that can be referenced against established normative values, offering objective insights into peripheral nerve function. In addition, electrophysiological parameters correlate with neuropathic pain, physical findings, and glycosylated hemoglobin levels [45].

A fundamental limitation of NCS lies in the fact that it predominantly assesses large myelinated fibers, whereas small unmyelinated or thinly myelinated fibers remain inaccessible to standard electrophysiological testing. As such, NCS may fail to detect early or small fiber–predominant neuropathic changes, underscoring the need for complementary diagnostic modalities.

### 5.2. Diagnostic Tools for Small‐Fiber Dysfunction in Diabetic Neuropathy

Emerging and specialized diagnostic tools enhance sensitivity in early or subclinical stages. Quantitative sensory testing (QST) provides psychophysical measures of thermal and pain thresholds, while epidermal nerve fiber density (ENFD) analysis via punch skin biopsy offers a histopathological index of small fiber degeneration [46] (Table 1).

In parallel, a number of emerging electrophysiological techniques have been developed to assess small‐fiber function beyond conventional NCS, which primarily reflect large‐fiber integrity. Methods such as contact heat–evoked potentials and laser‐evoked potentials enable objective evaluation of nociceptive Aδ‐ and C‐fiber pathways and have demonstrated sensitivity in early and painful forms of diabetic neuropathy [47]. Although currently confined largely to research settings, these approaches offer valuable mechanistic insight and may complement existing diagnostic tools as they become further validated. Finally, assessment of autonomic function is also essential. Techniques such as electrochemical skin conductance (Sudoscan) provide a simple and non‐invasive means to evaluate sudomotor dysfunction, thereby contributing valuable information for the diagnosis and characterization of DNs [48, 49].

Several non‐invasive imaging and surrogate markers have further expanded diagnostic capabilities. Corneal confocal microscopy (CCM) enables in vivo visualization and quantification of corneal nerve fibers and has emerged as a sensitive biomarker of small‐fiber pathology and regeneration [50]. Magnetic resonance neurography (MRN) allows high‐resolution visualization of peripheral nerve architecture and may facilitate more precise phenotyping of neuropathic alterations [51]; in the future, MRN could also provide robust imaging endpoints for clinical trials [52]. Additional emerging techniques are summarized in Table 1 [48, 49].

### 5.3. Magnetic Resonance Imaging

**Figure 3:**
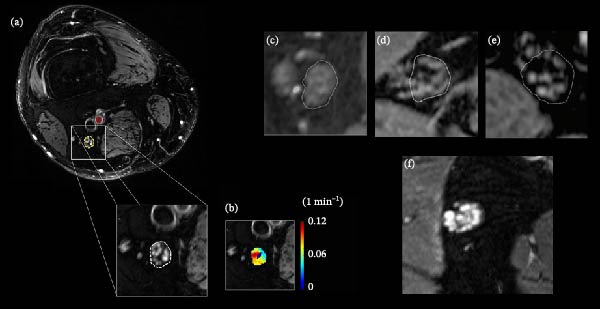
MRI in diabetic polyneuropathy. (a) T2‐weighted MR image of the distal right thigh showing the tibial portion of the sciatic nerve (yellow circle) and the adjacent femoral artery (red circle). (b) Color‐coded map of Ktrans values derived from the extended Tofts model, indicating sciatic nerve microvascular permeability,1 which is reduced in advanced disease. (c–e) Examples of sciatic nerve MRI in three different people with diabetes type 2 at various stages of diabetic polyneuropathy: (c) no polyneuropathy, (d) moderate polyneuropathy, and (e) severe polyneuropathy. (f) Sciatic nerve in a patient with type 1 diabetes and severe polyneuropathy, showing diffuse T2 hyperintensity across all nerve fascicles. Images adapted from 1 and 2 under a CC‐BY license.

MRN is a promising non‐invasive modality for assessing peripheral nerve structure in DPN, although its clinical applicability remains investigational. Advances in high‐field MRI (3T and 7T) enable fascicular‐level visualization and support the concept of a “virtual biopsy” [53–55] MRN combines T1‐weighted, fat‐suppressed T2‐weighted, and diffusion‐weighted imaging to provide high‐contrast views of nerve architecture. MRN lesion load correlates with DPN severity, with predominant involvement of the proximal sciatic nerve [55–57]. 3T MRI studies demonstrate marked fascicular alterations at the level of the sciatic nerve in the thigh, see also Figure 3 [57–60].

This proximal predominance may reflect limited detection of distal lesions by 3T MRN but also ischemic impairment of axonal transport causing axonal swelling [61]). The distal sciatic nerve, located between arterial supply zones, is particularly vulnerable to circulatory insufficiency (“watershed phenomenon”) [62]. MRN findings, including reduced microvascular permeability (Figure 3b), increased T2 signal, and enlarged interfascicular space (Figure 3c–e) may reflect ischemic nerve injury, potentially linked to fiber loss and intraneural fat accumulation. Differences between diabetes subtypes have been reported, with T1D‐associated damage linked to glycemic control and impaired conduction, and T2D to lipid metabolism alterations [60]. Figure 3f illustrates these differences, showing diffuse T2 hyperintensity in T1D without significant fiber enlargement, in contrast to severe T2D polyneuropathy (Figure 3e). These abnormalities mainly reflect dysfunction in medium and large myelinated fibers, with limited sensitivity to small unmyelinated C‐fiber pathology. Clinical use remains limited by lack of standardized protocols, uncertain pathophysiological correlates, and overlap with healthy individuals. Overall, MRN remains primarily investigational but offers a reproducible approach to assess peripheral nerve integrity with potential future applications.

### 5.4. Biomarkers Plasma

Numerous studies have explored the utility of plasma biomarkers for DPN, aiming to identify accessible indicators of disease progression [63]. These biomarkers span several mechanistic domains, reflecting the complex and multifactorial nature of DPN pathogenesis [64]. Inflammatory biomarkers include circulating cytokines such as tumor necrosis factor‐alpha (TNF‐α), interleukins (IL‐6, IL‐1β) [63], monocyte chemoattractant protein‐1 (MCP‐1), and transforming growth factor‐beta (TGF‐β), as well as acute‐phase proteins like glycoprotein acetylation (GlycA) and hematologic indices such as the neutrophil‐to‐lymphocyte ratio, all indicative of chronic inflammation [65]. Oxidative stress and endothelial dysfunction are captured by molecules such as heme oxygenase‐1 (HO‐1), glyoxalase I (GLO I), and glutathione‐dependent enzymes, highlighting redox imbalance and microvascular injury. Neuronal damage is particularly reflected by axonal and glial proteins measurable in plasma, among which neurofilament light (NfL) chain has emerged as a promising candidate [66]. NfL, a key cytoskeletal protein in large and myelinated axons, is released into the bloodstream following axonal degeneration [66]. In DPN, elevated plasma NFL levels have been correlated with clinical severity, nerve conduction abnormalities, and END loss, positioning NfL as a sensitive marker of neuroaxonal injury even in subclinical stages [67]. Its quantification through high‐sensitivity assays such as Simoa allows dynamic monitoring of axonal integrity over time. Nonetheless, its limited disease specificity being elevated in various neurodegenerative and inflammatory conditions suggests that NFL is best utilized in combination with other biomarkers, including neuron‐specific enolase (NSE), Schwann cell–associated proteins like TMPRSS5, and regulatory microRNAs (e.g., miR‐146a, miR‐29a), which contribute additional layers of information on neuronal stress, inflammation, and epigenetic dysregulation, and these observations are predominantly supported by preclinical and non‐human data [68]. Together, these biomarkers offer a multifaceted window into DPN pathology, though integrated approaches remain essential to enhance diagnostic precision and guide therapeutic strategies. Importantly, a distinction should be made between biomarkers with established clinical support, such as NfL chain, and those that are primarily supported by experimental or early‐stage studies, including several microRNAs, to better reflect their current level of clinical applicability.

## 6. Management

### 6.1. Current Standard of Care

DPN represents one of the most prevalent and debilitating complications associated with diabetes, affecting approximately half of people with diabetes over time. Its clinical manifestations range from asymptomatic nerve dysfunction to severe neuropathic pain and sensory loss, which predispose people with diabetes to foot ulcers and subsequent amputations. Effective management of DPN demands a comprehensive, stepwise strategy that combines both pharmacological and non‐pharmacological interventions, addressing symptom relief, prevention of progression, and improvement of patient quality of life.

The initial step in managing DPN focuses on early detection through systematic screening of at‐risk populations. Annual clinical evaluations using validated sensory tests such as the 10 g monofilament, tuning fork for vibration perception, or more advanced QST are essential to identify neuropathy at its incipient stages, even in the absence of overt symptoms. Early diagnosis permits timely initiation of therapeutic measures, which can mitigate the evolution of nerve damage.

Following diagnosis, stringent optimization of glycemic control becomes paramount, particularly in diabetes with type 1 diabetes where intensive glucose management has demonstrated significant reductions in neuropathic progression [69]. Following diagnosis, stringent optimization of glycemic control becomes paramount, particularly in diabetes with type 1 diabetes where intensive glucose management has demonstrated significant reductions in neuropathic progression [69]. In individuals with type 2 diabetes, multifactorial risk factor modification plays a crucial role. This involves not only glucose regulation but also rigorous management of hypertension, dyslipidemia, obesity, and smoking cessation. Such a holistic cardiometabolic approach is foundational to prevent further neuronal injury and attenuate disease advancement.

When neuropathic pain is present, pharmacological treatment should be introduced early to improve symptom control and enhance patient functionality. First‐line agents generally include serotonin‐norepinephrine reuptake inhibitors (SNRIs) like duloxetine and gabapentinoids such as pregabalin, both supported by robust clinical evidence demonstrating efficacy in reducing pain intensity. Tricyclic antidepressants, for example, amitriptyline, although effective, are usually reserved for younger individuals or those without significant cardiac comorbidities due to their adverse effect profile. In cases of localized pain, topical therapies including high‐concentration capsaicin patches or lidocaine may be utilized, particularly when systemic side effects pose concerns. Treatment should be personalized, and evidence suggests that combination therapy may improve analgesic efficacy compared with monotherapy with the potential, though not an inherent requirement, to allow lower doses of individual agents in selected people with diabetes, thereby reducing dose‐related adverse effects [70–72].

### 6.2. Emerging and Investigational Therapies

**Figure 4:**
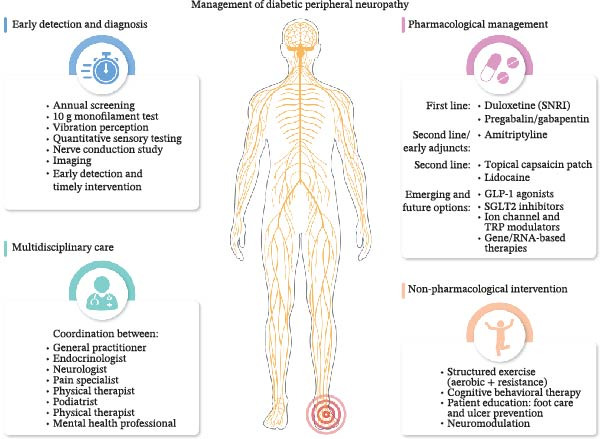
Management of diabetic peripheral neuropathy. Effective management of diabetic peripheral neuropathy involves early detection through screening and diagnostic tests, pharmacological treatments (e.g., duloxetine, pregabalin, topical agents), non‐pharmacological interventions (exercise, cognitive therapy, neuromodulation), and coordinated multidisciplinary care. Emerging therapies include GLP‐1 agonists, SGLT2 inhibitors, and gene‐based approaches.

In circumstances where first‐line pharmacotherapies do not provide sufficient relief or induce intolerable side effects, emerging options may be considered. Novel agents such as glucagon‐like peptide‐1 (GLP‐1) receptor agonists and sodium‐glucose co‐transporter 2 (SGLT2) inhibitors have shown potential neuroprotective effects beyond their metabolic benefits. Experimental therapies targeting specific ion channels (NaV1.7), transient receptor potential (TRP) channels, and inflammatory mediators are under investigation and may offer future disease‐modifying possibilities. Gene therapy and RNA‐based interventions (miRNA mimics or inhibitors, siRNA, antisense oligonucleotides) also represent promising avenues for neuropathic pain management, though they remain in the early research phase (Figure 4).

Simultaneously, non‐pharmacological interventions are integral to comprehensive DPN care. Structured exercise regimens combining aerobic and resistance training have been demonstrated to improve nerve conduction and reduce neuropathic symptoms. Psychological therapies, notably cognitive behavioral therapy (CBT), provide important support for people with diabetes coping with chronic pain and associated mood disorders [73]. Additionally, education on foot care and protective measures is vital to minimize the risk of ulceration and amputation. For refractory pain, neuromodulation techniques such as transcutaneous electrical nerve stimulation (TENS) or spinal cord stimulation may be warranted [74].

Ultimately, the management of DPN necessitates a personalized, multidisciplinary approach that integrates metabolic control, pharmacotherapy, and lifestyle modifications. Coordination between endocrinologists, neurologists, pain specialists, physical therapists, and mental health professionals ensures that treatment plans are adapted to individual patient needs, optimizing outcomes. Through such a systematic and holistic strategy, it is possible to alleviate neuropathic pain, slow disease progression, and significantly enhance individual’s quality of life.

## 7. Conclusion

DPN represents a major and growing complication of diabetes, affecting both type 1 and type 2 populations, and increasingly observed in prediabetes. Despite significant advances in understanding its multifactorial pathogenesis, ranging from metabolic and vascular dysfunction to inflammatory and neurodegenerative mechanisms, its diagnosis remains complex, especially in early stages. NCS and emerging imaging modalities such as MRN offer valuable diagnostic insights, further research is warranted to better appreciated their complementary in both diagnostic and severity assessment. Current management relies on glycemic optimization, symptomatic pharmacotherapy, and integrative lifestyle interventions, yet no definitive disease‐modifying treatment exists to date. Future research should prioritize longitudinal studies, personalized therapeutic strategies, and biomarker validation to improve early detection and outcomes. A multidisciplinary approach remains essential to reduce the burden of DPN and enhance quality of life for affected individuals.

## Funding

This work was supported by the Swiss National Science Foundation (#10005333). Open access publishing facilitated by Universite de Geneve, as part of the Wiley ‐ Universite de Geneve agreement via the Consortium Of Swiss Academic Libraries.

## Conflicts of Interest

The authors declare no conflicts of interest.

## Data Availability

Data sharing not applicable to this article as no datasets were generated or analyzed during the current study.
